# Supplementary material for: Effects of a High-Protein Diet on Kidney Injury under Conditions of Non-CKD or CKD in Mice
Source: Int J Mol Sci. 2023 Apr 24;24(9):7778. doi: 10.3390/ijms24097778 (PMC10177820; doi:10.3390/ijms24097778)
Supplement: Supplementary file 1 [file ijms-24-07778-s001.zip › Supplementary Figure S1.pptx]

## Slide 1
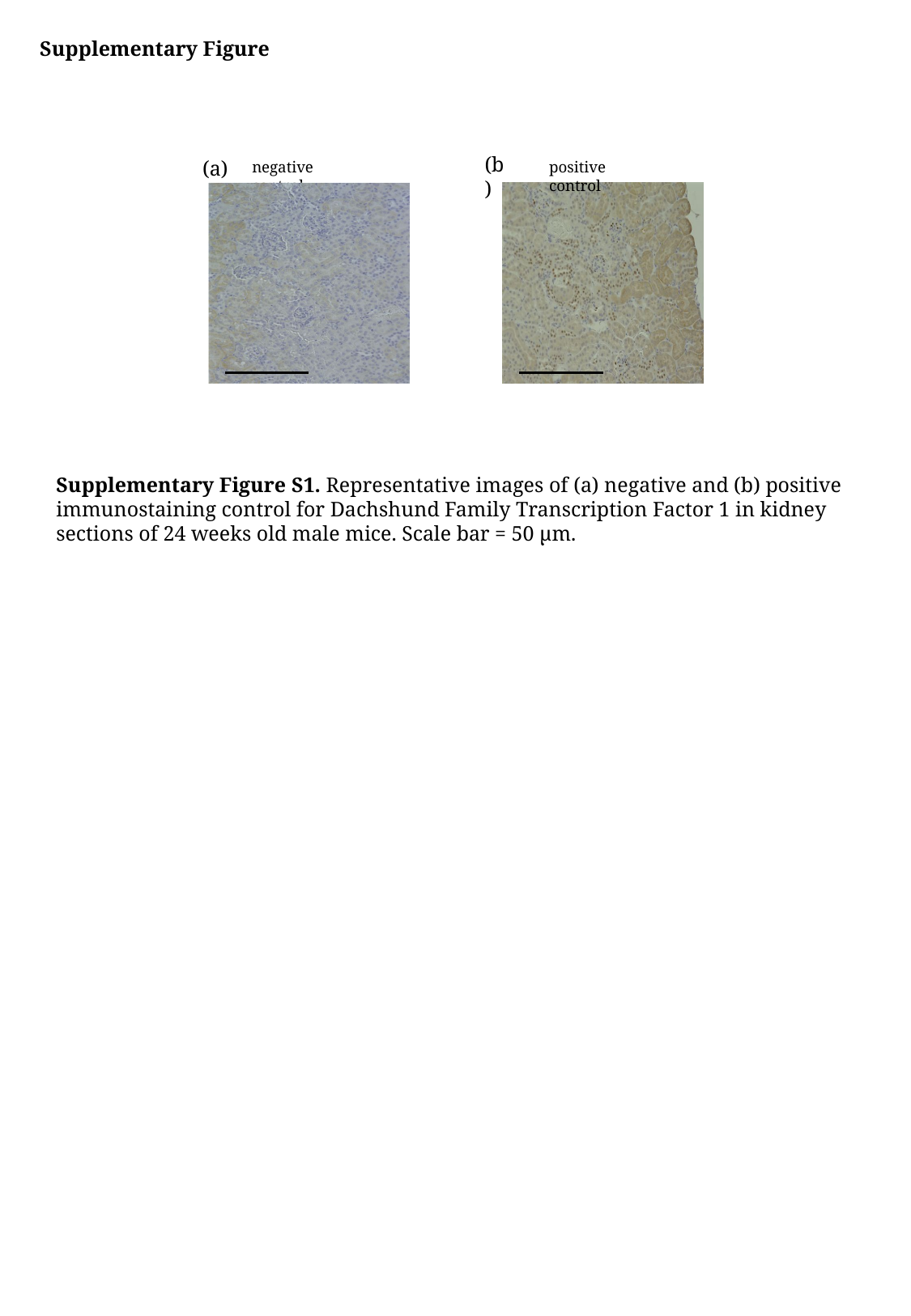

Supplementary Figure
(b)
(a)
negative control
positive control
Supplementary Figure S1. Representative images of (a) negative and (b) positive immunostaining control for Dachshund Family Transcription Factor 1 in kidney sections of 24 weeks old male mice. Scale bar = 50 µm.
