# Supplementary material for: Effects of a High-Protein Diet on Kidney Injury under Conditions of Non-CKD or CKD in Mice
Source: Int J Mol Sci. 2023 Apr 24;24(9):7778. doi: 10.3390/ijms24097778 (PMC10177820; doi:10.3390/ijms24097778)
Supplement: Supplementary file 1 [file ijms-24-07778-s001.zip › Supplementary Figure S2.pptx]

## Slide 1
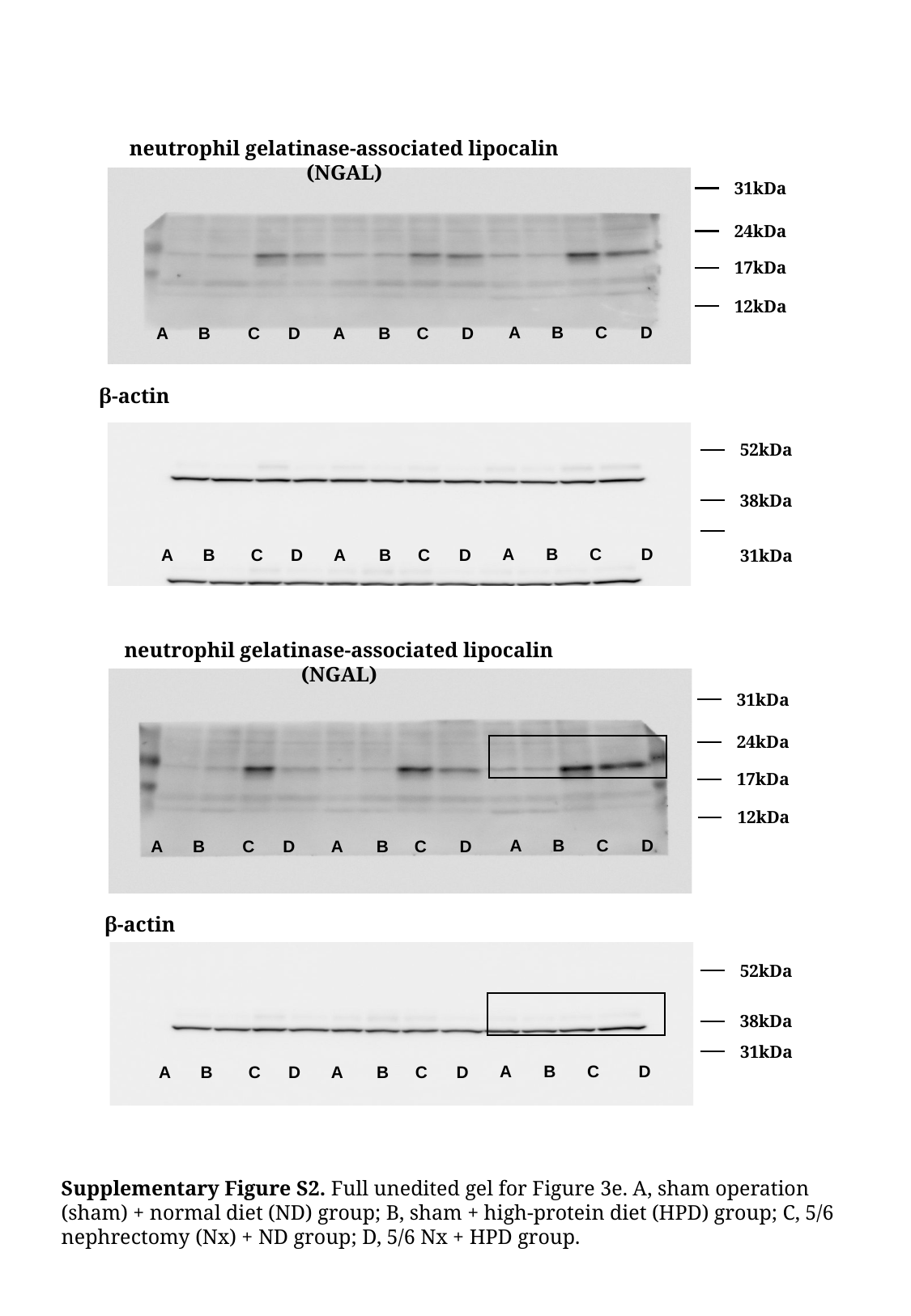

neutrophil gelatinase-associated lipocalin (NGAL)
31kDa
24kDa
17kDa
12kDa
B
A
D
C
B
B
A
D
C
D
A
C
β-actin
52kDa
38kDa
B
A
D
C
B
B
A
D
C
D
A
C
31kDa
neutrophil gelatinase-associated lipocalin (NGAL)
31kDa
24kDa
17kDa
12kDa
B
A
D
C
B
B
A
D
C
D
A
C
β-actin
52kDa
38kDa
31kDa
B
A
D
C
B
B
A
D
C
D
A
C
Supplementary Figure S2. Full unedited gel for Figure 3e. A, sham operation (sham) + normal diet (ND) group; B, sham + high-protein diet (HPD) group; C, 5/6 nephrectomy (Nx) + ND group; D, 5/6 Nx + HPD group.
